# Numerical Investigation of the Defects Effect in Additive Manufactured Ti-6Al-4V Struts on Deformation Behavior Based on Microtomographic Images

**DOI:** 10.3390/ma15144807

**Published:** 2022-07-09

**Authors:** Michał Doroszko

**Affiliations:** Department of Mechanics and Applied Computer Science, Faculty of Mechanical Engineering, Bialystok University of Technology, 45C Wiejska, 15-351 Bialystok, Poland; m.doroszko@pb.edu.pl

**Keywords:** deformation process, finite element analysis, additive manufacturing, lattice structures, microtomography

## Abstract

This paper describes the influence of defects occurring in struts under tension, obtained using the additive method of laser powder bed fusion (LPBF), on the stress and strain distributions. The study used struts of different thicknesses separated from Ti-6Al-4V diamond lattice structures. For numerical modeling of stress and strain fields, models that reflect the realistic shape of the tested struts with their imperfections were used. The shape of the diamond structure struts was obtained based on microtomographic measurements. Based on the results obtained, the influence of defects in the material structure on the stress and strain distribution was analyzed. It was observed that the main factor influencing the stress and strain distribution in the struts are micronotches on their external surface. These imperfections have a significantly greater impact on the stress and strain concentration than the micropores inside. Furthermore, the interactions of the imperfections are also important, which in turn affects the stress distributions and the formation of bands of high-stress values inside the material. The relationship between the presence of micropores, the stress–strain curves, and the mechanical properties of the material was also assessed.

## 1. Introduction

Due to rapid developments in advanced biomedical structures and the materials used, additive methods for metal materials are becoming more and more important in production [1,2]. The specificity of these production methods enables the production of complex geometries and structures that would be difficult to obtain with other methods. The construction of elements from structures allows for a significant reduction in the weight and stiffness of the designed elements [3,4]. However, it also negatively affects their strength. Therefore, research aimed at obtaining the appropriate mechanical properties required in a given case is very important [5]. On the other hand, understanding how the technological imperfections of individual struts affect their mechanical properties is necessary for modifying the production process in order to reduce the impact on the deformation process and material strength [6].

Numerical calculations and computer simulation tests allow a deeper understanding of the deformation process of materials such as lattice structures obtained by 3D printing [7]. They also allow for assessing the impact of imperfections of these types of materials resulting from the specificity of the layer-by-layer production process. Many methods are currently used to account for the imperfections in lattice structures and other heterogeneous metallic materials. These methods mainly involve obtaining cross-sections of the tested materials. Then, based on the cross-sections, the three-dimensional shape of the examined structure is recreated [8]. Two main approaches can be distinguished, one of which is based on attempts to generate an imperfect shape of material structures from the data of the structure [9,10,11,12]. The second approach is to reproduce the shape as realistically as possible based on a material specimen [13,14,15,16]. In the case of the method based on realistic mapping, computer microtomography (micro-CT) is most frequently used [17,18]. This method allows non-destructive, relatively easy, and quick material cross-sections to be obtained in the form of tomographic images, compared to other serial sectioning methods that usually use microscopy to record the cross-section view [19]. Using the technique of computed microtomography, it is possible to recreate the three-dimensional geometry of material structures along with their defects [20,21]. Including imperfections in individual struts and nodes in lattice structures allows a more accurate simulation of the material deformation process than the highly simplified models [11,22,23,24]. In the case of lattice structure struts produced using additive methods, the imperfections of the structure resulting from the technological process are oversize thickness, micronotches between the folds of the material on the external surface, and internal closed micropores. All of these defects significantly affect the deformation process of the material and its mechanical properties.

This paper describes the numerical modeling of the tensile process of the lattice structure struts of the Ti-6Al-4V alloy, produced with the use of the LPBF additive method intended for the 3D printing of metals [25]. The influence of the defects formed in the struts of the mesostructure on its deformation process was investigated. The described studies are a continuation of the considerations contained in the work of Doroszko et al. [26]. Microtomographic measurement was used to take into account the complicated shape of the external surfaces of the struts and the internal microporosity. Based on the tomographic images, the realistic shape of the examined structures was recreated. To carry out a detailed analysis of the effects of the imperfections on the deformation process of the material, single struts of the lattice structure from the three-dimensional geometric models were separated. In the calculations using the finite element method, the nonlinearity of the material was taken into account using the true stress–strain curve determined for the Ti-6Al-4V material with a relative density close to 1 (porosity close to 0), which was obtained in the same way as the tested specimens of the mesostructures. Based on the performed calculations, the stress and strain distributions in the tensile deformed struts were obtained. The influence of the shape of the external surface and internal micropores on the obtained values and the places of stress and strain concentrations were determined. The nominal stress–strain curves, taking into account the minimum, average, and maximum values of the cross-sectional area of the struts and the corresponding mechanical properties of the material, were also obtained. Based on an analysis of the results of the calculations, the influence of the local defects of the strut’s shape on their properties on a macroscopic scale was described. It was also determined how the imperfections of the material’s mesostructure can influence the initiation of material fracture.

## 2. Materials and Methods

For the research in this paper, Ti-6Al-4V titanium alloy, which is used in many biomedical applications due to its high biocompatibility, was used. The raw material was LaserForm Ti Gr23 powder (3D Systems) with a particle size of a few to 40 µm. The specimens intended for investigation were printed using the LPBF method and the 3D Systems ProX DMP 320 device. In the present study, diamond lattice structures were used, which are widely considered and used as metamaterials for bone-replacement components as they are strongly isotropic [27]. This makes them suitable for the production of biomedical implants, where loads in different directions usually occur. The diamond structure is also well-suited for production by direct metal printing (DMP) because there are no struts with a longitudinal axis parallel to the horizontal plane, which helps to reduce the amount of dross [28]. Diamond structures were produced with a relative density of 18.5% and 27% for the structure struts with a nominal diameter of 0.49 and 0.6 mm, respectively. The main production parameters are a layer thickness of 60 µm and a scanning speed of 400 mm/s. Other printing parameters were optimized by the Medgal^®^ Orthopedic Implants and Instruments specimen manufacturer. The materials produced were heated in a vacuum oven at a temperature of 920 °C to remove residual stress.

The realistic shape of the struts of the diamond structures was taken into account in numerical modeling. Single struts were separated based on microtomographic images of entire diamond structures. Microtomographic measurements were made using a Bruker SkyScan 1172 device. Computed microtomography was performed with a pixel size of 2.94 µm, a current of 100 μA, and a source voltage of 100 kV. In this way, most of the details of the geometry of the examined structures, which may have an impact on the deformation process of the material, were recreated. Based on the obtained tomographic images, the shape of struts of the concerned structures was mapped. For this purpose, Avizo 9.7.0 software (Thermo Scientific, Waltham, MA, USA) was used. For mapping the shape based on microtomographic images, a procedure analogous to that described in the work by Doroszko et al. [26] was used:

- Layering of tomographic images (cross-sections) and definition of their orientation in the software.
- Binarization of the images using thresholding to separate the shape of the diamond structure from the tomographic images.
- Separation of struts from diamond structures.
- Triangulation of the struts’ exterior surfaces and interior pore surfaces based on generated voxel layers.
- Generation of surface finite element mesh.
- Conversion to solid finite element mesh.

**Figure 1:**
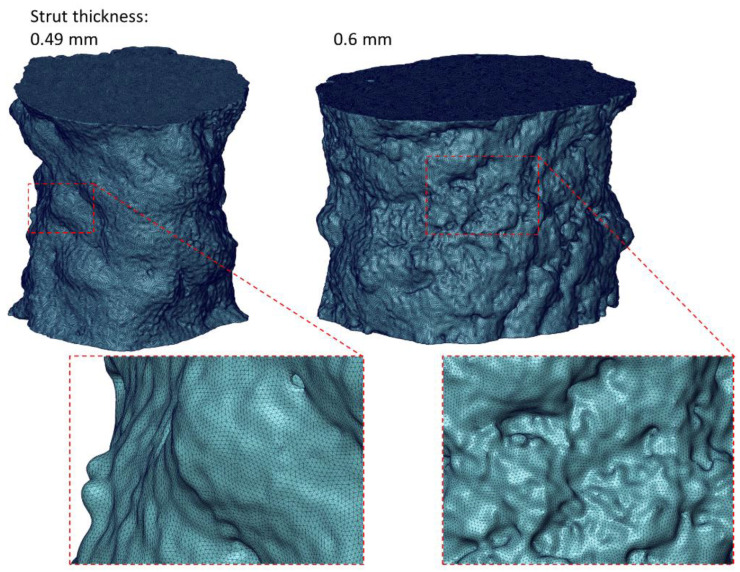
Division into finite elements of the examined material structures.

**Figure 2:**
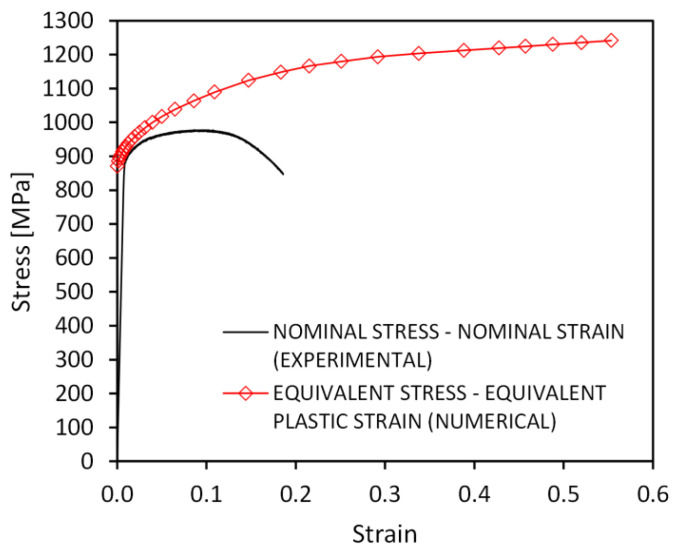
The true and nominal stress–strain curves for Ti–6Al–4V solid material obtained using 3D printing [26].

In this work, the deformation process of the diamond structure struts was analyzed using the finite element method. The Marc software (MSC Software) used for the advanced nonlinear calculations was used for the numerical calculations. The shapes of the struts generated based on microtomographic images were imported in the form of surface finite element meshes. Then, they were converted into solid finite element meshes. Four-node tetrahedral solid finite elements of type Tetra 134 [29] with an average size of approximately 0.003 mm were used for modeling. Such a size of finite elements will significantly improve the mapping of the smallest details of the strut geometry compared to previous research, where the size of the finite elements was about 0.02 mm [26]. For a strut with a thickness of 0.49 mm, a mesh of 5,308,535 finite elements was generated, and for a strut with a thickness of 0.6 mm, a mesh of 5,382,676 finite elements was generated. Figure 1 shows the finite element meshes generated for both struts. In the numerical calculations, the elastic–plastic material model with the Huber-von Mises plasticity criterion and isotropic hardening was used. Young’s modulus of 116.9 GPa and Poisson’s ratio of 0.31 were obtained for the Ti-6Al-4V material with a relative density close to that of the solid material, which was prepared in the same way as lattice structures. The nonlinearity of the material was taken into account in the calculations using the true stress–strain curve (equivalent stress-equivalent plastic strain), which was also determined for a material close to solid using the experimental-numerical method [30,31]. It is an iterative method in which, at first, a nominal stress–strain curve is used in the numerical calculation as the nonlinearity of the material, taking into account only the initial values of the cross-sectional area of the specimen and the gauge length. Then, the force–displacement curve is read and compared with the experimental curve. Based on an analysis of their discrepancy, a modification of the stress–strain curve is performed and recalculation is carried out. Iterations are carried out until satisfactory agreement is obtained between the numerically and experimentally obtained force–displacement curves. In this way, the stress–strain relationship was taken into account for the entire range of plastic strain up to the moment of fracture initiation. Figure 2 shows a comparison of the true and nominal stress–strain curves. The large discrepancy between the curves and the much larger values of stress and strain in the true curve is due to inclusion in the numerical calculations of material nonlinearity, the change in cross-sectional area, and the inhomogeneous distribution of stress and strain in the neck of the specimen resulting from tensile deformation. In order to simulate the tension of the struts, boundary conditions were used, which consisted of a zero displacement of the nodes on the lower base of the model and the displacement of the nodes on the upper base in the direction of the strut axis (z-direction). The applied value of the nodal displacement allowed for the elongation of the struts to a maximum of 5%. Tensile simulations of the struts were carried out under quasi-static conditions at an initial strain rate of 0.0004 [1/s], at which the properties and nonlinearity of the near-solid material were determined, as in previous experimental and numerical studies [26].

## 3. Results and Discussion

Based on the performed numerical calculations, stress and strain distributions were generated in deformed struts of diamond structures. Based on these results, the ways in which the irregularities of a structure’s shape and microporosity affect the material deformation process as a result of tension were determined. The basic mechanical properties and nominal stress–strain curves for the struts both with and without the internal closed pores were also determined.

### 3.1. Defect Analysis Based on Micro-CT

**Figure 3:**
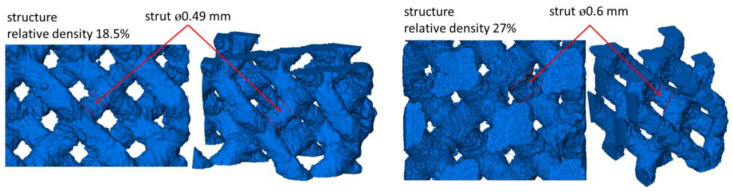
Additive manufactured Ti-6Al-4V diamond structures used for numerical modeling of the deformation process of the struts.

**Figure 4:**
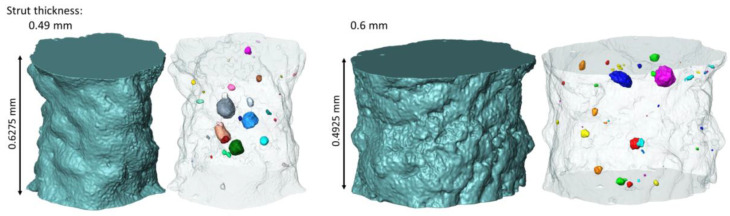
Surfaces of the struts and internal micropores recreated based on microtomographic images.

**Figure 5:**
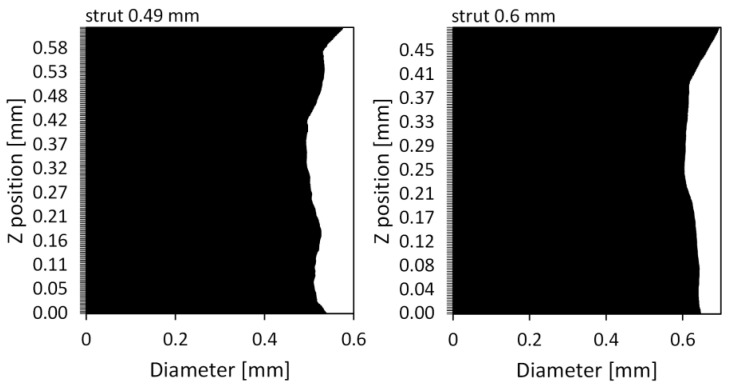
Graphs of the changes in the equivalent diameter of the struts at their height.

**Figure 6:**
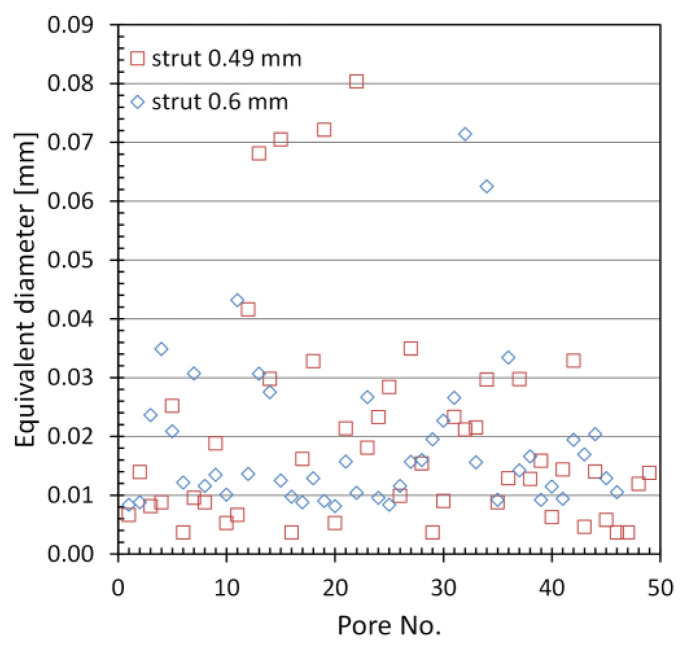
Equivalent diameter distribution of closed micropores in the investigated struts.

**Figure 7:**
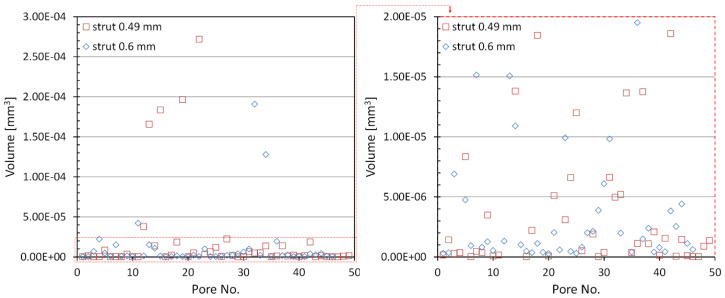
Volume distribution of closed micropores in the investigated struts.

Figure 3 shows the entirety of scanned structures and indicates the places from which the struts for numerical modeling were separated, whereas Figure 4 shows the shape of the separated struts and the micropores closed inside them. It can be seen that the surface of a strut with a nominal thickness of 0.49 mm is much less corrugated than the surface of a beam with a nominal thickness of 0.6 mm. Based on the two-dimensional cross-sections of the investigated struts, the ways in which the dimension of the equivalent diameter *D*_eq2D_ of a strut changes in relation to its height was also examined. The equivalent diameter was measured using Avizo 9.7.0 software successively for individual cross-sections of the investigated struts based on microtomographic images. In the 2D images, the equivalent diameter is measured as the diameter of a disk with the same area as the considered cross-section [29]:

(1) $$D_{\mathrm{eq}2D}=\sqrt{\frac{4A}{\pi }}$$

where *A* is the strut cross-section area. In Figure 5 it can be seen that the measured diameters are oversized in relation to the nominal value over the entire height of the struts. In the structures examined, the overestimated value of the diameter of the struts amounts to an average of 5.1% and 4.7% for struts with a nominal diameter of 0.49 and 0.6 mm, respectively. However, the maximum difference of the measured dimension from the nominal is 17.1% for a strut with a thickness of 0.49 mm and 15.5% for a strut with a thickness of 0.6 mm. Based on the presented diagrams of the measurements of the strut diameters (Figure 5), it can be expected that the variability of their dimensions will have an impact on the process of their deformation as a result of tension, and in places where the diameters change the fastest, the stress will be concentrated. In addition, an analysis of the closed micropores inside the materials was performed. For this purpose, the equivalent diameter and volume of the individual pores closed in the struts were also measured with the Avizo 9.7.0 software. In the case of measuring the equivalent diameter of pores, they were referred to as their three-dimensional representation. In the 3D images, the equivalent diameter *D*_eq3D_ is measured as the diameter of a sphere with the same volume as the considered pore [32]:

(2) $$D_{\mathrm{eq}3D}=\sqrt{\frac{6V}{\pi }}$$

where *V* is the pore volume. Figure 6 shows the equivalent diameter distribution of closed micropores in the concerned struts. In both cases, there is a small number of pores with diameters ranging from 60 µm to 80 µm (4 pores for a 0.49 mm strut and 2 for a 0.6 mm strut), whereas the majority of pores have diameters in the range of 4–16 µm. It should also be noted that the 0.49 mm diameter strut has a significant number of small pores in the range of 3.5–10 µm (38.8% of the pores). However, in the case of a strut with a diameter of 0.6 mm, the diameter of the majority of the smallest pores is between 8 and 16 µm (60.9% of the pores). Both structures contain single larger pores with volumes ranging from 125,000 to 275,000 µm^3^, whereas most are in the range of 100 to 2500 µm^3^ (Figure 7). The number of pores measured in the considered struts was comparable in both cases and at 49 and 46, respectively, for structures with a nominal thickness of 0.48 and 0.6 mm.

### 3.2. Stress and Strain Fields in Struts

**Figure 8:**
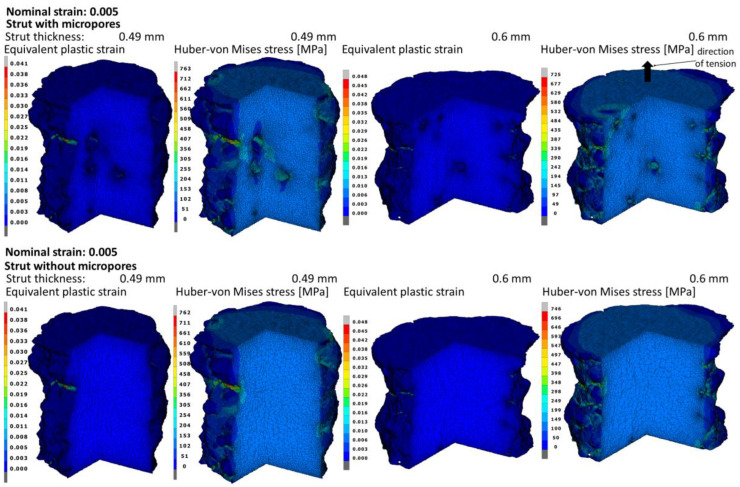
Equivalent plastic strain and Huber–von Mises stress fields in the struts with and without micropores at the moment of obtaining the nominal strain of 0.005.

Figure 8 shows the distributions of the equivalent plastic strain and stress according to the Huber–von Mises hypothesis for the macroscopic strain of 0.005 with and without pores. Based on the plastic strain distributions, it can be seen that the maximum values obtained are about 10 times higher than the nominal strain value. The highest values of the stress and strain under consideration were obtained on the external surface of the strut in the recesses forming technological micronotches on the surface where the initiation of the plastic deformation of the material takes place. The abovementioned recesses are the result of inaccuracies in the production process as well as its nature, i.e., the element structure made layer-by-layer of powder with defined parameters. On the surface of micropores, there are also higher values than the mean values in volume, but they are much lower than those on the surface.

**Figure 9:**
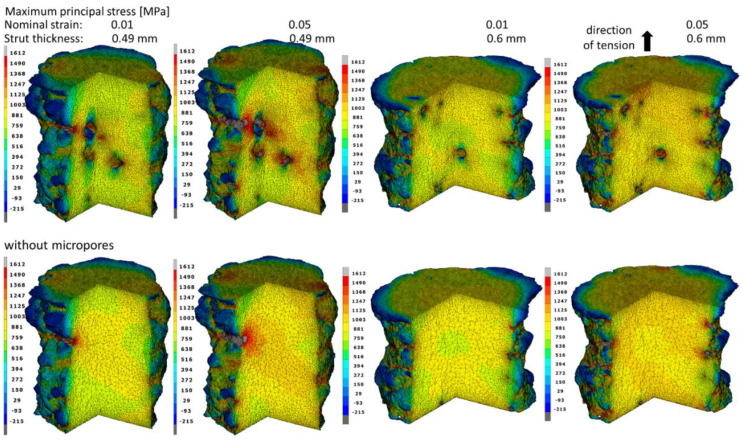
Maximum principal stress fields in the struts with and without micropores at the moment of obtaining the nominal strains of 0.01 and 0.05.

**Figure 10:**
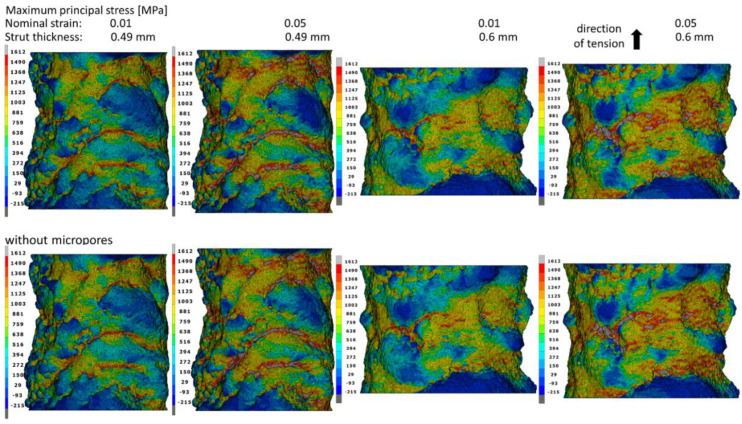
Maximum principal stress fields on the struts’ surfaces with and without micropores at the moment of obtaining the nominal strains of 0.01 and 0.05.

The distribution of the maximum principal stress for the nominal deformations of 1% and 5% in struts with and without pores was also analyzed (Figure 9). Based on the research carried out in the work of Doroszko et al. [26], it was determined that the critical value of the maximum principal stress for the tested material was *σ*_c_ = 1612 MPa. When this value is exceeded, fracture initiation occurs in the material. For this reason, it is the maximum value in the principal stress distributions. In the case of struts elongated by 1%, the stress value close to the critical value occurs both in the recesses on the outer surface and the surface of the inner micropores, whereas for a nominal deformation of 5%, the critical stress in these places was exceeded, which may indicate a local fracture initiation of the material. In models with micropores, tension results in more extensive zones of high-stress values than in the case of struts without pores due to the interaction of external and internal stress concentrators. Due to the smaller cross-sectional area of 0.49 mm-thick struts, the influence of porosity on the stress values is much greater than in 0.6 mm bars where the distances between individual pores are much larger. In the case of larger diameter struts, there is also a small interaction between the external and internal stress concentrators. This means that the presence of internal micropores weakens the material less than it does in a 0.49 mm-thick strut. Figure 10 summarizes the maximum principal stress distributions on the outer surface of the bars. On the outer surfaces of struts elongated by 5%, there are significantly more and larger zones in which the value of the critical stress is exceeded than in the case of the pore surface. This means that the undulating shape of the external surfaces of the struts has a much greater impact on the initiation of material fracture than the interaction of the micropores. The places with the highest values are arranged in bands in the recesses created as a result of the layer-by-layer production of the material. Therefore, it should be assumed that improvements in the surface quality by smoothing it as a result of changes in the material production process may cause an increase in the range of nominal strain in which no fracture of the material occurs. Figure 10 shows that taking into account the porosity of the material has a negligible effect on the stress values of the external surfaces of both struts.

**Figure 11:**
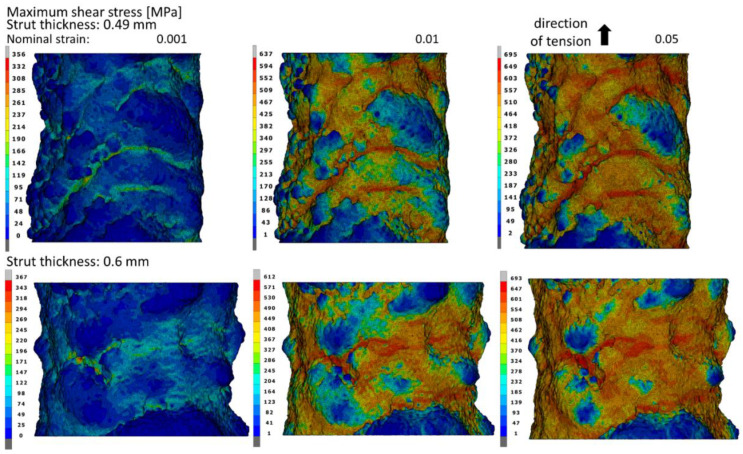
Maximum shear stress fields on the struts’ surfaces with micropores at the moment of obtaining the nominal strains of 0.001, 0.01, and 0.05.

Figure 11 shows the maximum shear stress distributions in the struts elongated by 0.1%, 1%, and 5%. The places where the maximum values of the shear stress were obtained are very similar to the zones of the maximum principal stress. The maximum shear stress values obtained of 695 MPa and 693 MPa for 0.49 mm- and 0.6 mm-thick struts, respectively, are significant in relation to the values of the maximum principal stress. For this reason, it should be noted that in addition to the maximum principal stress, the obtained values of shear stress can also have a large influence on the fracture initiation of the material. For both struts’ thicknesses, the maximum values of the shear stress are comparable over the entire range of the considered nominal strain, and the difference amounts to a maximum of less than 4%.

### 3.3. Mechanical Properties

As part of the work, the basic macroscopic mechanical properties of the tested struts and the nominal stress–strain curves were also determined. In Table 1, the values obtained for Young’s modulus and yield stress for struts with and without micropores are summarized. In addition, the variability of the cross-sectional area along the length of the struts was also taken into account, and the properties were calculated for the minimum, mean, and maximum values of the equivalent diameter of the struts. The mean difference between the minimum and maximum values of Young’s modulus and yield stress is about 35%. The significant discrepancy is related to the high surface irregularity of the considered structures, whereas the minimum values are approximately 19% lower and the maximum values are 9% higher than the determined average values of Young’s modulus and yield stress. The determined average value of Young’s modulus in a strut with a thickness of 0.49 mm without porosity was 1.9% higher and for a strut with a thickness of 0.6 mm it was 0.8% higher than for the structure with microporosity. However, in the case of the average yield stress in the strut without pores, the values were 3.4% and 1% higher, corresponding to structures with a thickness of 0.49 and 0.6 mm.

**Figure 12:**
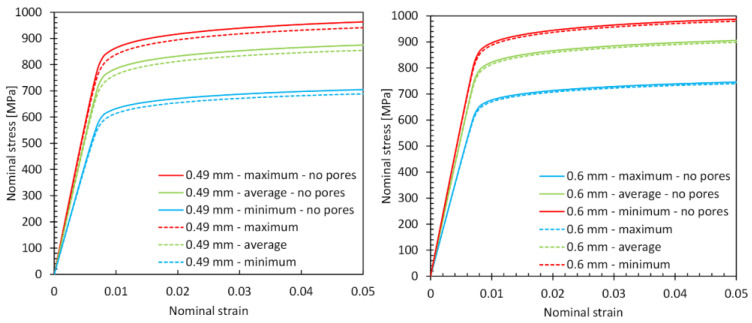
Nominal stress–strain curves obtained for 0.49 mm (on the (**left**)) and 0.6 mm (on the (**right**)) strut diameters under tension with a nominal strain of up to 0.05.

The character of the discrepancy in the values of the mechanical properties is also recreated in the nominal stress–strain curves (Figure 12). It can also be seen that the differences between the curves for porous and nonporous struts are higher for a 0.49 mm strut than for a 0.6 mm strut, where the differences are negligible. This indicates a negligible influence of micropores on the macroscopic deformation of a 0.6 mm-thick strut. The reason for this is the greater distances between the individual pores, which as a result of tension do not have such a large interaction with each other as in the case of a strut with a smaller cross-sectional area, and thus do not cause such a large difference in the stiffness of the structure. It should also be noted that the levels of nominal stress and the shape of the curves obtained for both the investigated struts are similar, regardless of their thickness. The nominal stress values in larger diameter struts are slightly higher.

## 4. Conclusions

In this work, the deformation process of individual struts separated from the Ti-6Al-4V diamond lattice structure obtained by the additive LPBF method was investigated and described. Due to the use of microtomographic measurements in the research, details of the geometry resulting from the production process, such as recesses and folds on the external surface of the struts and internal closed micropores, were reproduced. The geometric imperfections mentioned above significantly affect the deformation process of the material as a result of its tension. Based on the numerical calculations performed, the stress and strain distributions in the investigated material were obtained. Macroscopic mechanical properties and nominal stress–strain curves were also determined. When the obtained results were analyzed, the extent to which individual groups of structure defects affect the material deformation process was indicated.

The main factor influencing the deformation process as well as the distribution of stress and strain in the tensioned struts are defects on their external surfaces. They are bands of folds and recesses created as a result of building the structure layer by layer. In the recesses between successive folds, technological micronotches are formed and embedded in the surface, in which the highest concentration of stress and strain takes place. The second type of structure defect is internal closed micropores on the surface of which there is also a stress concentration. The obtained maximum stress values on the pores’ surfaces are typically much lower than the external notches. It was also noticed that in a 0.49 mm-thick strut, the distances between the single pores are small and thus there are interactions between them and bands with high stress values. This effect is much smaller in the 0.6 mm-thick beams, where the pore spacing is much larger due to the larger cross-section. In addition to the direct influence of the structures’ defects described above, the interaction of the imperfections is also important as it affects the stress distributions. The high stress values in the recesses of the surfaces of the struts near the pores result in the formation of stress zones in which fracture initiation can take place.

Based on the analysis of the maximum principal stress distributions, it can be concluded that material imperfections can initiate fracture. The high values of the maximum shear stress in places where the highest values of the principal stress also occur, indicate the presence of a multiaxial stress state in these places. For this reason, local material fracture modeling should be performed using fracture criteria that take into account the complex stress state [33]. In addition, based on the distribution of stress and strain in a realistic structure, it is possible to perform analyses of the fatigue properties of the material. The stress and strain concentrations caused by micronotches on the external surface with micropores inside the struts significantly reduce the fatigue life of the structure, especially in the area of high-cycle fatigue. This will be considered as a future research topic aimed at an even better understanding of the processes related to the deformation and fracture of the investigated materials.

The conducted research is also the basis for the modification of the technological process aimed at limiting the influence of micronotches and micropores resulting from the specificity of the laser powder bed fusion method. Additional research should be carried out on the influence of the parameters of the production process and the obtained shape of the struts on the concentration of stress and strain in the material. In this way, the strength and fatigue life of the material could be improved and increased.

## Figures and Tables

**Table 1:** Minimum, average, and maximum nominal values of Young’s modulus and yield stress.

Strut Thickness [mm]	*E*_min_ [GPa]	*E*_av_ [GPa]	*E*_max_ [GPa]	$\sigma_{y}^{\min}[\mathrm{MPa}]$	$\sigma_{y}^{\mathrm{av}}[\mathrm{MPa}]$	$\sigma_{y}^{\max}[\mathrm{MPa}]$
0.49	83.4	105.4	114	607	754	831
0.49 (no pores)	84.9	107.6	116.1	629	778	858
0.6	88.6	107.5	117.3	668	809	882
0.6 (no pores)	89.2	108.4	118.2	672	818	893

## Data Availability

Not applicable.
